# *Cannabis*-Based Oral Emulsion for Medical Purposes to Meet the Needs of Patients: Formulation, Quality and Stability

**DOI:** 10.3390/pharmaceutics14030513

**Published:** 2022-02-25

**Authors:** Francesca Baratta, Elena Peira, Carola Maza, Marina Gallarate, Paola Brusa

**Affiliations:** Department of Drug Science and Technology, University of Turin, Via Pietro Giuria 9, 10125 Turin, Italy; elena.peira@unito.it (E.P.); carola.maza@unito.it (C.M.); marina.gallarate@unito.it (M.G.); paola.brusa@unito.it (P.B.)

**Keywords:** medical *Cannabis*, *Cannabis* oil, THC, CBD, standard procedures, stability

## Abstract

Current Italian legislation provides that medical *Cannabis* can be administered orally as an extract if it has been titrated to determine the concentration of active molecules. In this context, there is a need to provide known and adequate quantities of active ingredients in order to guarantee uniform therapies that lead to the optimization of risks/benefits. This is fundamental considering that the limited availability on the market of registered *Cannabis*-based products for medical use means that prescribed therapies are usually prepared as galenic preparations. Consequently, the preparation procedures must be consistent with the instrumentation usually present in the laboratories of community pharmacies. In this context, the purpose of this work was to standardize the preparation procedure for oil-in-water (O/W) emulsions to exploit advantages in terms of ease of administration and dosage adjustment, but also to ensure the palatable organoleptic characteristics of the finished product. For the formulations being studied, in addition to the quality according to the directives set out in the European Pharmacopoeia, the stability was evaluated to assure adequate validity for therapeutic uses.

## 1. Introduction

The earliest records of *Cannabis* being used for medical purposes date back to 4000 BCE in China. Thereafter, its use spread throughout Asia, the Middle East and Africa. The plant came to the attention of Western scientists in 1839 when William O’Shaughnessy, a British medical doctor and surgeon working in India, discovered its potential. In the 20th century, the situation mutated, and *Cannabis* was stigmatised for the abuse of its psychotropic effects, and this led to it being banned from commerce and made illegal in many countries [1,2].

However, in recent years, there has been a notable increase in the prescription of *Cannabis* for medical purposes; this is, in part, thanks to the support of the media, as well as the high expectations for its efficacy. However, this has yet to be fully backed up by valid scientific research [3,4].

Data on the efficacy of *Cannabis* for medical use are insufficient and inadequate for several reasons: among these is the poor design of the studies that have been conducted, and the scarcity of standardised *Cannabis* preparations suitable for research. Moreover, in recent years, a large part of the literature and research funding has centred on its potentially harmful effects, rather than its use for medical purposes. However, the interest that this latter topic has stimulated is evident when reviewing the growing number of articles published and patents deposited that aim to evaluate the actual medical efficacy of this plant and its components [5,6].

Currently, a limited number of cannabinoid-based products have been authorised for sale: Marinol^®^ (AbbVie Inc., North Chicago, IL, USA), which contains dronabinol, a synthetic isomer of delta-9-tetrahydrocannabinol; Cesamet^®^, which contains nabilone (Meda Pharmaceuticals Inc., Somerset, NJ, USA), a synthetic cannabinoid; and Sativex^®^ (GW Pharma Ltd., Cambridge, UK) containing an ethanol extraction of *Cannabis sativa*. In addition to these, pharmaceutical-grade inflorescences have become available: compared with synthetic products, these natural forms have far greater potential for use in medicine. They contain the complete range of active molecules that make up the plant phytocomplex. This is fundamental in terms of the hypothesised synergistic mechanism between cannabinoids and terpenes known as the “Entourage effect”. The phytocomplex of *Cannabis* is made up of as many as 500 different molecules, of which approximately a hundred belong to the cannabinoid chemical class, and, among these, even minor differences in structure may induce very different effects [7,8].

*Cannabis* inflorescences for medical use have therefore offered physicians, in those countries where it is legal, the opportunity to prescribe magistral formulae based on *Cannabis*. The inflorescences come from a number of varieties with different concentrations of active molecules: on this subject, it is important to stress that the concentrations of THC and CBD indicated on the label must be inferred as referring to the “total” content: the sum of the molecule in acid form (delta-9-tetrahydrocannabinolic acid—THCA- and cannabidiol acid—CBDA-) and decarboxylated form (delta-9-tetrahydrocannabinol—THC- and cannabidiol—CBD-). The molecules of greatest pharmaceutical interest are, however, those in the decarboxylated form, as these are most easily absorbed in the intestine [9]. The inflorescences, which may be sold in the raw state or prior to grinding, can be vaporised in specifically designed devices, or can undergo extraction to obtain compounds suitable for oral administration [2,5,10].

In the above context, Italy has introduced legislation on the use of *Cannabis* for medical purposes and authorised its cultivation at the Stabilimento Chimico Farmaceutico Militare (Military Chemical Pharmacological Facility) in Florence, which has been selling a type of *Cannabis*, called FM2, since 2016. This is supplied as dried and ground inflorescences with a total THC concentration between 5% and 8%, and a total CBD concentration between 7.5% and 12%. In 2018, another variety of *Cannabis* called FM1 also became available, containing a total THC concentration between 13.0% and 20.0%, and a total CBD concentration below 1% [11,12,13].

The current legislation in Italy allows the administration of medical use *Cannabis* in oral or in inhalable forms. Administration of *Cannabis* for medical use by inhalation is considered a secondary choice [12,14].

In accordance with the Ministry of Health Directives, the decoction is the preferred oral pharmaceutical form, prepared in accordance with the official procedure reported in the document “Raccomandazioni per il medico prescrittore di sostanza vegetale *Cannabis* FM-2 infiorescenze” (Recommendations for physicians prescribing vegetable substances FM2 *Cannabis* inflorescences) [14]. However, as we demonstrated in a previous study carried out with decoctions of FM2 *Cannabis* inflorescences, this type of procedure should be avoided due to the low yields of recovered THC and CBD, the high volume of the decoction that the patient would therefore have to ingest, and the high raw material costs [10].

Concerning oral administration, current regulations provide that medical *Cannabis* may also be administered as an extract, provided that this has been titrated to determine the concentration of active molecules [12]. Although the administration of consistent known quantities of active molecules is essential to assure therapies for which it is possible to conduct a proper evaluation of efficacy, the current regulations in Italy do not indicate clearly which molecules must be titrated: the standard practice is to titrate THC and CBD. On this last point, it is important to stress that, while a number of extraction methods have been reported in scientific literature for the production of magistral preparations based on medical *Cannabis*, there is no official standard preparation procedure. Hence, the galenic preparations obtained are generally very different in terms of the titrated concentration of active molecules. This variability stems from the application of differing operating protocols during the preparation phase, but also because of the natural variability of *Cannabis* inflorescences. To minimise the former factor, it is to be hoped that there will soon be a standard production protocol for *Cannabis* extracts [5,15,16,17,18].

To fill this gap, in 2018, we developed a novel medical *Cannabis* extraction method for FM2 strains in olive oil (called β-4). The procedure was compared with the most widely used extraction methods in Italy at the time. The comparative tests showed that the β-4 method was particularly efficacious, with final concentrations of decarboxylated THC and CBD significantly higher than those obtainable with water-based extraction (decoction) and with oil extraction in accordance with the then existing methods [10]. Having optimised the extraction procedure, stability studies were performed which demonstrated the stability of the oil under different environmental conditions over 180 days. To facilitate the assumption of the prescribed therapy by the patient, we then developed a standard procedure for a single-dose preparation in capsules containing the β-4 oil. The quality and stability of this preparation was then evaluated. Oils have, unfortunately, particularly unpleasant organoleptic properties and this may affect adherence to therapy. The encapsulation of the oils has the advantage of masking the organoleptic characteristics and, hence, encouraging a significantly greater adherence to therapy among patients enrolled in any research project [19].

Of course, it is necessary to bear in mind that administering pharmaceuticals in solid form is not suitable for all patients because of the difficulty in ingesting them, as well as the challenges in adjusting the dosage, should it become necessary.

In light of these considerations, the purpose of this work was to standardise the preparation procedure for a liquid form suitable for oral use from β-4 oils. In particular, various types of oil-in-water (O/W) emulsions were developed to exploit the advantages cited above in terms of ease of administration and dosage adjustment, but also to ensure the palatable organoleptic characteristics of the finished product. For the formulations being studied, in addition to the quality according to the directives set out in the European Pharmacopoeia [20], the stability was evaluated to assure adequate validity for therapeutic uses.

## 2. Materials and Methods

### 2.1. Active Compounds

All of the galenic preparations described below were based on flowering tops from type FM2 *Cannabis* purchased from the Pharmaceutical Chemicals Military Facility in Florence. The titrated concentrations of active compounds in the unprocessed material were 2.54 ± 0.33% for THC, 2.97 ± 0.41% for THCA, 1.71 ± 0.26% for CBD, 6.29 ± 0.72% for CBDA. Consequently, the total THC, calculated by the formula %THC tot = %THC + (0.877 × %THCA), was 5.14 ± 0.69% and the total CBD content, calculated by the formula %CBD tot = %CBD + (0.877 × %CBDA), was 7.23 ± 0.95%. The method β-4 allows us to obtain oils (*Cannabis* β-4 oils) with an average concentration of active molecules in the decarboxylated form of 8.04 mg/mL for THC and 13.05 mg/mL of CBD [10].

### 2.2. Oils and Emulsions Preparation 

For the preparation of the *Cannabis* oil, a precise quantity of FM2 *Cannabis* inflorescences was immersed in a precise quantity of extra-virgin olive oil in a weight to volume ratio of 200:1 (mg/mL). The oil containing the inflorescences was then placed in a water bath at boiling point with a stirrer for 60 min. Subsequently, the oil was filtered using a cotton gauze or a hydrophilic cotton gauze in a manual press. Before the oil extraction phase, the *Cannabis* flowers had been ground for 60 s to produce a uniformly sized batch; spread in a thin layer (5 mm max; optimal thickness 1–2 mm) and placed in an oven at 140 °C for 30 min. The temperature applied was based on the fact that this is close to the evaporation point of THC (145 °C) [21]. The volume of the batches prepared was between 5 and 100 mL [10].

Using the oils obtained with the described procedure, three different types of O/W emulsion were prepared. Their composition is shown in Table 1. The materials used for the galenic preparations were purchased from a pharmaceutical supplies company (Farmalabor s.r.l, Canosa di Puglia, Bari, Italy) and complied with the relevant monograph of the European Pharmacopoeia. 

All types of emulsion were prepared both manually—emulsions 1, 2 and 3- and with the aid of a turbo-emulsifier (IKA-T25 digital Ultraturrax)—emulsions 1T, 2T and 3T-. In particular, 1 and 1T emulsions were prepared as follows: sodium methyl parahydroxybenzoate, poloxamer 407, glycerol and water-soluble lemon flavour were added to depured water. The mixture was left overnight at 4 °C and then added under manual stirring to the mixture of *Cannabis* β-4 oil and tocopherol. Both 2 and 2T emulsions were prepared in the same manner with a variation: soy lecithin was added to the mixture of *Cannabis* β-4 oil and tocopherol. In the case of emulsion 3 and 3T, sodium methyl parahydroxybenzoate was solubilized in depured water; the obtained mixture was added under manual stirring to the *Cannabis* β-4 oil. At the end of the preparation process, T emulsions were turboemulsified.

### 2.3. Quantitative Analysis

For the evaluation of the uniformity of the emulsions content according to the directives set out in the European Pharmacopeia [20], a standard procedure was developed starting from the method applied by Alvarez-Fuentes et al. [22] THC and CBD levels were assessed through a HPLC method using a UV detector (YL9300 liquid chromatograph, Younglin Instruments Co., Anyang, Korea). All chemicals were of analytical grade (Sigma–Aldrich, Milan, Italy). Experiments were performed in the absence of direct sunlight at room temperature (15–25 °C). Analytical chromatographic conditions were: Zorbax Eclipse XDB-C18, 80 Å, 4.6 × 250 mm, 5 µm column; a mobile phase consisting of two solvents: a mixture of acetonitrile:water:acetic acid (A) [75:23.7:1.3 *v*/*v*] and acetonitrile (B). Eluent (70%:30%) was pumped at a flow-rate of 1 mL/min. The detection wavelength UV was 275 nm [23] and the injection volume was 20 µL. The elution time was approximately 6 min for CBD and 12 min for THC. After each analysis the column was washed with 100% acetonitrile for 15 min and then brought back to the initial conditions with the mobile phase.

Calibration curves were built using either THC and CBD analytical standard solutions or β-4 oils at concentrations, ranging from 0.1 and 10 mg/mL for both THC and CBD. All the samples to be analysed were diluted with isopropanol to obtain a final concentration suitable for the range of the calibration curve. In particular, in order to quantify the amount of THC and CBD in the emulsions, a quantity of 100 µL of isopropanol were added to 20 mg of the preparation to be analysed, the sample was vortexed (Velp Scientific ZX3 vortex machine) for 60 s and then centrifuged (Beckman Coulter Microfuge 18 Centrifuge) at 10,000 rotations per minute for 30 min. The supernatant thus obtained was separated, suitably diluted to fall within the range of the calibration curves and analysed as described above.

### 2.4. Evaluation of Emulsions Stability

The emulsions were tested at regular intervals in order to evaluate the stability after storage at room temperature. In particular, the following parameters were assessed: the uniformity of content using the method described in the previous Section 2.3, the pH (Hanna HI 9321 pH meter AC/DC input 110 V), and any phase separation following centrifugation at 3000 rpm for 30 min (Rotofix 32 centrifuge, Hettich Italia srl, Milan, Italy). The droplet shape and size were observed using an optical microscope (LEICA DM 2500 microscope, Leica Microsystems GmbH, Wetzlar, Germany) connected to a digital camera at ×630 magnification. The microscope images were analysed using the Motic Images 2000 software. For the samples, the diameter was measured for a minimum of 100 droplets and the mean diameter was calculated. The listed tests were performed immediately after the preparation of the emulsion and then repeated every 7 days for 28 days.

Rheological behaviour and viscosity measurements were performed with a Brookfield rotational rheometer/viscometer (DV-III+, Brookfield, Milwaukee, WI, USA) with spindle coupled to a temperature-controlling unit. The samples were thermostated with a 25 °C circulating bath connected to the viscometer. Viscosity was recorded at fixed shear rates. The rheological behaviour was estimated by submitting the samples to increasing and then to progressively decreasing shear rates in an appropriate range for each system. Rheograms were constructed by plotting shear stress as a function of shear rate. The rheological behaviour and viscosity measurements were performed immediately after the preparation and then again after 28 days. 

All tests were repeated in triplicate and on three different lots for each type of emulsion. 

The samples were stored in dark containers and protected from light. For each parameter, the average of the obtained results was evaluated, and the corresponding standard deviation (SD) was calculated. Values were accepted if SD was less than 2%. The samples under analysis were stored protected from light.

## 3. Results

Each β-4 oil emulsion type was analysed immediately after preparation using the method described in Section 2.3. The results confirmed that the concentration of active molecules in each emulsion was consistent, regardless of the composition. HPLC analysis revealed that the variation in concentration of THC and CBD in the different emulsion batches was less than 10%. In addition, no variation in the expected concentration greater than 10% was detected after 28 days, except for emulsion type 3. In this case, phase separation was evident in this emulsion and, while it was possible to disperse the oil phase again by agitation, the concentration of the active molecules of interest was inconsistent because of the non-uniform dispersion of the oil phase in the aqueous one. 

The microscope examination revealed that all the emulsion types prepared using an Ultraturrax disperser in the preparation phase were characterised by a greater homogeneity in droplet dispersion. Emulsion 2T had the best emulsion characteristics as the droplets had the smallest size and the tightest particle size distribution. Droplets mean diameters are reported in Table 2. It was not possible to measure the droplet size for the 3 emulsions at T28 due to phase separation.

**Figure 1:**
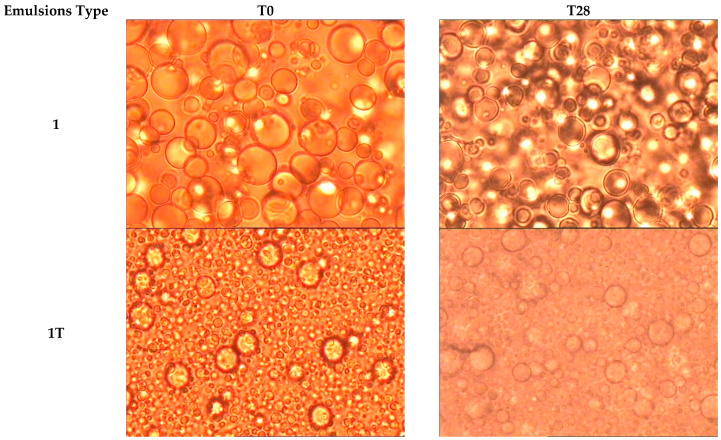

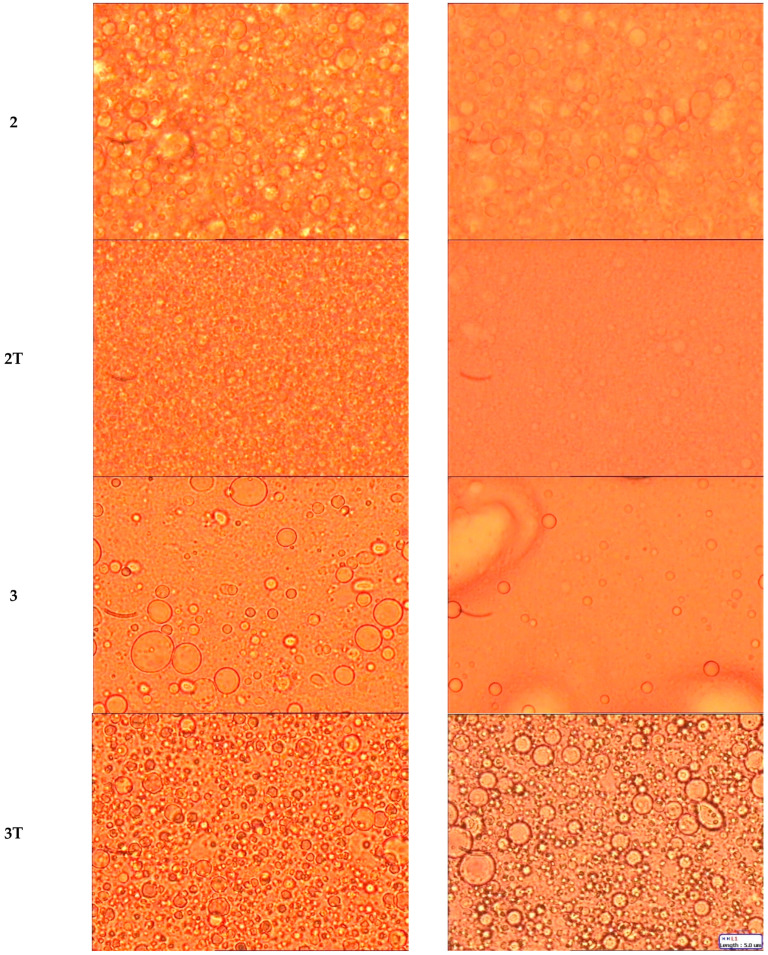
Emulsion micrographs at T0 and T28 days (at ×630 magnification).

The stability over time of the dispersed drops in the emulsions was evaluated (see Figure 1). It was found that there were no significant changes in droplet dimension for the emulsion types 1, 1T, 2, and 2T after 28 days stored at room temperature. However, after 28 days, there was no longer any evidence of droplets in emulsion 3: this is probably due to the phase separation that had occurred, which also prevented us from obtaining a homogenous sample. The dimension of the droplets in emulsion 3T had increased in size, and were clearly unstable, but phase separation had not yet occurred.

The viscosity of each emulsion was measured at a shear rate (SR) of 0.02 s^−1^, apart from emulsion 3T, which, given the low viscosity of the samples, had to be measured at a SR of 25 s^−1^. Table 3 reports the average viscosity for each emulsion type. There is a clear difference in the viscosity values taken at T0 and T28 for all the manually emulsified preparations (variation > 20%). Conversely, the viscosity remained constant over time for the emulsions that had been mechanically emulsified in the preparation phase, with a variation of less than 10%. It was not possible to measure viscosity for emulsion 3 at T28 due to phase separation.

Rheological tests were performed on samples of all the emulsions, but only emulsion 2 and emulsion 2T had characteristics of rheological interest, therefore, data obtained for emulsions 1 and 3 are not reported.

**Figure 2:**
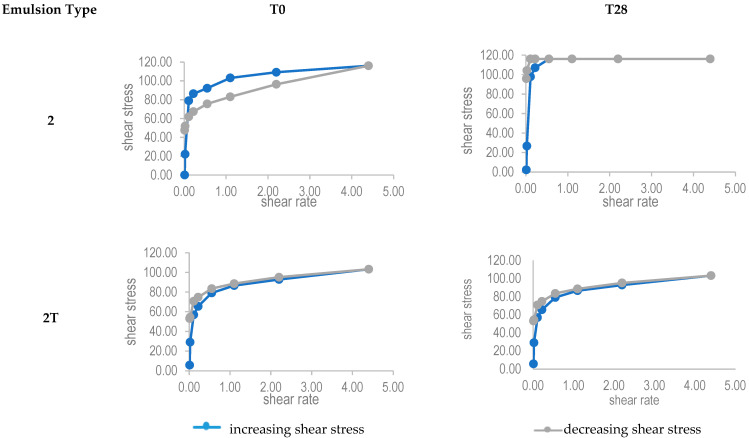
Rheological behaviour of emulsions 2 and 2T.

As shown in Figure 2, both emulsions 2 and 2T displayed non-Newtonian behaviour at T0. Observing the curve obtained at increasing shear stresses, both emulsions showed pseudoplastic behaviour. At T0, emulsion 2 showed time-dependent viscosity characterized by a thixotropy hysteresis loop, which was not present in emulsion 2T, which showed no-time dependent viscosity for high shear rates. Thixotropy, however, is not required in a formulation for oral administration. 

Concerning pH, Table 4 reports the average pH values of the emulsions. From the data, it can be observed that only emulsions 3 and 3T underwent a relevant variation in pH overtime. 

Centrifugation at 3000 rpm for 30 min caused the phase separation of emulsion 3 after storage at room temperature for 28 days. None of the other samples displayed any change following the same treatment.

The reference chromatograms for THC and CBD analytical standards, β-4 oil and 2T emulsions (Figure S1) and the results obtained from the quantitative analyses of the 2T emulsions (Table S1) have been reported in the Supplementary Material.

## 4. Discussion

In 2018, our research group developed a novel oil extraction method (called β-4 method) for medical *Cannabis*. Subsequently, it was compared with three other standard methods widely used in Italy. The β-4 method proved to be highly efficacious in that it yielded average concentrations of THC and CBD more than double those obtained with the methods used up to that point [10]. Having optimised the extraction method, we carried out studies over 180 days that demonstrated the stability of this product, not only for the oil extract itself, but also for a single-dose capsule form under various environmental conditions [19].

As evidenced in the scientific literature, oral forms of *Cannabis*-based therapies are a significantly less effective in terms of bioavailability than those taken by inhalation (5–20%). The pharmaceutical effect lasts from 30 min to 3 h and the maximum concentration of cannabinoids in the blood is usually reached after 2 h. Nevertheless, the oral route is generally preferred given the ease of administration [24,25,26,27]. The formulations developed during our research could, therefore, represent a promising new option in therapeutic situations. Although the capsule form has obvious advantages in that it masks the characteristic organoleptic properties of oil extracts of *Cannabis*, the objective of the present research was to develop a liquid form for oral administration. The disadvantage of a liquid form is its reduced long-term stability compared with solid preparations, even with the addition of preservatives. [28] However, this drawback is far outweighed by its suitability for subjects suffering from dysphagia, and the ease in adjusting dosage when necessary.

In this light, three formulations, were prepared using commonly available excipients widely used for galenic preparations in Italy. Furthermore, the preparation method was designed to be as simple as possible, also by providing for manual preparation, considering that the developed standard operating procedure must be easy to replicate in the compounding laboratory of any community or hospital pharmacy.

The test results for the three types of emulsions revealed that emulsions 3 and 3T, whose composition was undoubtedly the simplest among the preparations developed, were not suitable for administration. After storage at room temperature for 28 days, microscope examination revealed significant alterations in the dimension of the dispersed droplets that, as far as the manually emulsified solutions are concerned, led to a phase separation visible to the naked eye and confirmed by centrifugation thereafter. Moreover, after 28 days, emulsion 3 was no longer consistent in concentration of active molecules, and the pH both of emulsion 3 and emulsion 3T had changed considerably. Both emulsions contained only lecithin as emulsifier, and probably it was not sufficient to stabilize the system. So, the overtime decrease in pH was probably due to the tendency to phase separation, which led to structural changes in the system and in the location of lecithin at the interface. Finally, the reduced viscosity would make these formulations unpalatable.

Instead, emulsions 1, 1T, 2, and 2T showed no significant changes in the shape, dispersion, and dimension of the droplets, nor in pH, or concentration of active molecules throughout the timeframe of the test. In addition, centrifugation had no effect on these emulsions. The dimension of the emulsified droplets in batches 2 and 2T was visibly finer than that of the droplets in emulsions 1 and 1T. In particular, the samples of emulsion 2T had the smallest and most homogenous droplets. Regarding the viscosity of each emulsion, significant changes (>20%) could be detected after 28 days in storage only for the manually emulsified type 1 and type 2. The turbo-emulsified types had no significant alterations in viscosity. Probably, the mechanical emulsification, forced the internal structure of the emulsions to organize itself in a less deformable manner, confirmed by the lack of hysteresis areas in emulsions 2T. In emulsion 2T, no changes in the rheological behaviour were then recorded either over the 28-day duration of the tests; thus, confirming the stability of its internal structure.

Emulsion 2T is, therefore, characterised by droplets with the smallest diameter. Moreover, no significant alterations were detected in this emulsion for any of the parameters applied in the study for the entire duration of the test. As well as retaining its internal structure, demonstrated by rheological tests, emulsion 2T confirmed its excellent long-term stability. Consequently, this formulation is certainly the most suitable among all the preparations tested in this study. Probably, the greater homogeneity of the drops in the 2 and 2T emulsions and their smaller dimensions are to be related to the presence of a more complete emulsifying system. The addition of a surfactant agent such as lecithin and a rheological additive, e.g., Poloxamer 407, leads to a more compact film at the O/W interface, thus, further stabilising the final emulsion.

## 5. Conclusions

The lack of commercially available, approved *Cannabis*-based medication inevitably means that, for the most part, the prescribed therapy must be prepared galenically. Consequently, the preparation procedure must be congruous with the equipment commonly available in a community or hospital pharmacy’s compounding laboratory. For this reason, we chose to focus our research on the development of emulsions with a very simple composition and a simple preparation procedure, while bearing in mind the quality of the finished product, which must comply with current regulations. What is clear from the results obtained is that the fundamental step is mechanical dispersion. Though most pharmacies in Italy are not equipped with one, a turbo-emulsifier is fundamental to obtain the correct degree of droplet dispersion, dimension, and consistency; moreover, viscosity values of mechanical dispersed emulsions were subjected to minor variation overtime than manual dispersed ones. Certainly, this aspect must be considered by the pharmacist when equipping a compounding laboratory. However, this piece of equipment is not prohibitively expensive, costing the same as an analytical balance or a capsule filler; equipment that is normally present in any galenic laboratory. 

Due to the natural variability of *Cannabis* inflorescences, despite the good results achieved for β-4 oils in terms of concentrations of active constituents, unfortunately it is not possible to abolish the oil titration procedure, also taking into account that this is currently required by Italian law. The dosage of emulsion to be administered—through measuring spoons or graduated syringes—needs, therefore, to be calculated according to the medical prescription following the titration process. Certainly, the availability on the market of an already titrated *Cannabis* extract would make its preparation and titration superfluous with undoubted advantages for patients who could access the therapy more quickly.

In this project, we did not carry out any test specifically, nor are there any examples in the literature of exhaustive tests to assess any loss of components in the phytocomplex during the various preparation phases of the *Cannabis* extracts. Generally, despite the growing interest in the “Entourage” effect, testing concentrates on the quantification of THC, CBD, any terpenes, and a small number of other components. It seems essential to us to go deeper into this aspect of the question in future studies, to assess which components of the phytocomplex are actually responsible for any therapeutic activity in medical *Cannabis*.

## 6. Patents

An Italian patent was granted by the Italian Office for Patents and Brands for the procedure for *Cannabis* oil production (patent number 102018000011128, 17 November 2020).

## Figures and Tables

**Table 1:** Emulsions composition.

	Emulsion Type		
Ingredients	1 and 1T	2 and 2T	3 and 3T
*Cannabis* β-4 oil	10 g	10 g	10 g
Poloxamer 407	15 g	10 g	/
Soy lecithin	/	10 g	10 g
Glycerol	10 g	10 g	/
Sodium methyl parahydroxybenzoate	0.1 g	0.1 g	0.1 g
Tocopherol	0.05 g	0.05 g	0.05 g
Water-soluble lemon flavour	1 g	1 g	1 g
Depured water	q.s. to 100 g	q.s. to 100 g	q.s. to 100 g

**Table 2:** Mean diameters ± SD (µm) measured at T0 and T28.

Emulsion Type	T0	T28
	Mean Diameter ± SD (µm)	Mean Diameter ± SD. (µm)
1	7.70 ± 4.15	8.10 ± 3.18
1T	3.81 ± 1.98	3.82 ± 1.26
2	3.90 ± 1.40	4.41 ± 1.26
2T	2.12 ± 0.64	2.26 ± 0.78
3	5.81 ± 3.00	/
3T	3.39 ± 1.12	7.57 ± 2.05

**Table 3:** Viscosity values.

Emulsion Type	T0	T28	
	Mean Viscosity Values (mPa s)	Mean Viscosity Values (mPa s)	Δ% Compared to T0
1	1300.523	993.388	−23.62
1T	1218.940	1276.528	4.72
2	1002.986	1214.141	21.05
2T	1295.724	1319.718	1.85
3	44	/	/
3T	10.96	11.03	0.64

**Table 4:** pH values.

Emulsion Type	T0	T28	
	Mean pH Values	Mean pH Values	Δ% Compared to T0
1	8.26	7.95	−3.75
1T	9	8.86	−1.56
2	7.44	7.26	−2.42
2T	7.53	6.94	−7.84
3	7.37	6.22	−15.60
3T	7.42	5.6	−24.53

## Data Availability

Data is contained within the article.

## Supplementary Materials

The following supporting information can be downloaded at: https://www.mdpi.com/article/10.3390/pharmaceutics14030513/s1, Figure S1: Reference chromatograms for THC and CBD analytical standards, β-4 oil and 2T emulsions; Table S1: results obtained from the 2T emulsions quantitative analyzes.

## Abbreviations

CBD	cannabidiol
CBDA	cannabidiolic acid
O/W	oil-in-water
q.s.	quantum sufficit
SD	standard deviation
T0	initial conditions
T28	28 days of storage at room temperature
THC	delta-9-tetrahydrocannabinol
THCA	delta-9-tetrahydrocannabinolic acid
