# Supplementary material for: Cannabis-Based Oral Emulsion for Medical Purposes to Meet the Needs of Patients: Formulation, Quality and Stability
Source: Pharmaceutics. 2022 Feb 25;14(3):513. doi: 10.3390/pharmaceutics14030513 (PMC8954602; doi:10.3390/pharmaceutics14030513)
Supplement: Supplementary file 1 [file pharmaceutics-14-00513-s001.zip › pharmaceutics-1544895-supplementary.pdf]

pharmaceutics

MDPI

Figure S1 - Reference chromatograms for THC and CBD analytical standards, β-4 oil and 2T emulsions

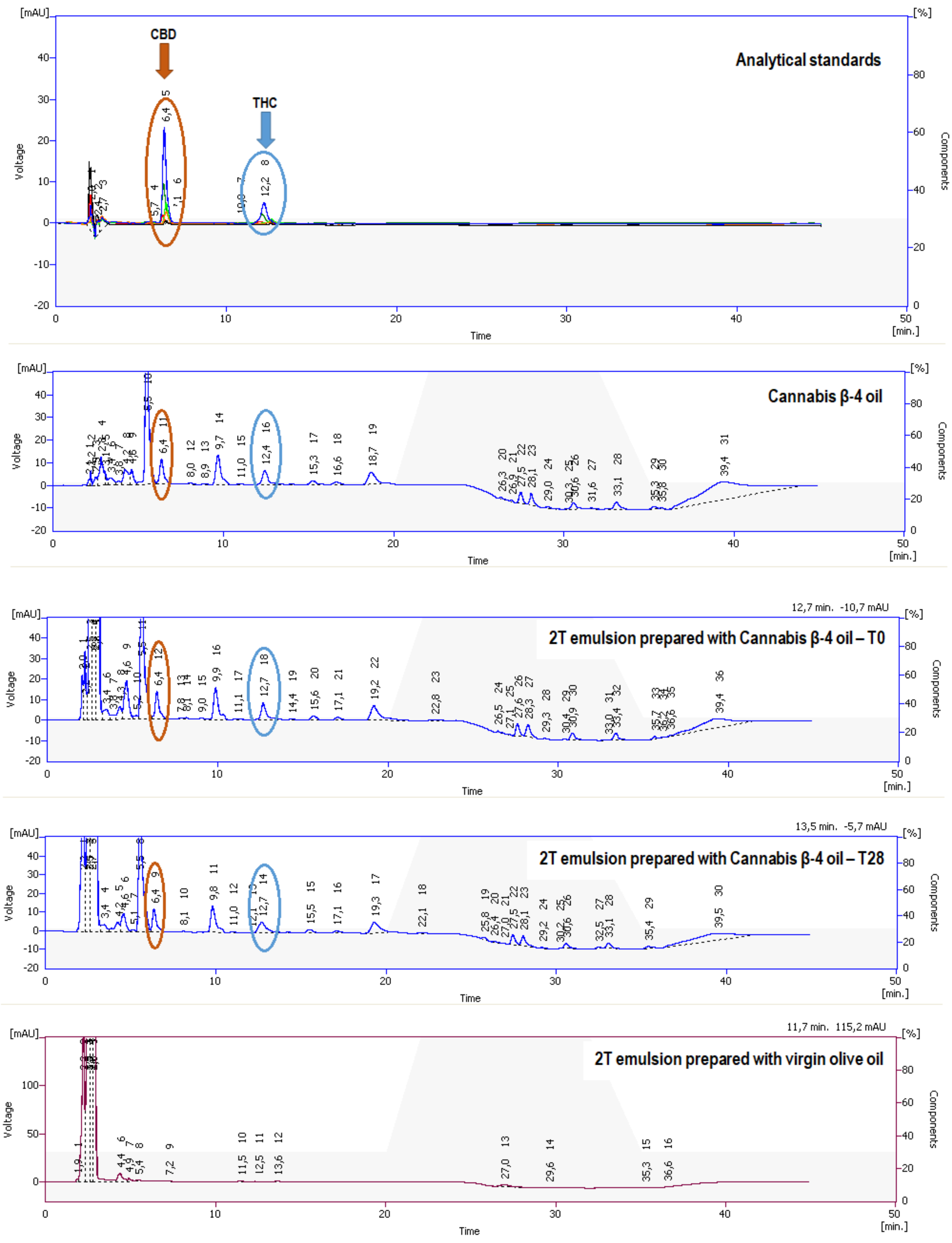

Table S1 - results obtained from the 2T emulsions quantitative analyzes

	2T EMULSIONS											
	THC						CBD					
	THC expected concentration in the emulsions (mg/g)	THC experimental concentration in the emulsions (mg/g)	mean value	SD	percentage variation compared to the mean value	percentage variation compared to the expected value	CBD expected concentration in the emulsions (mg/g)	CBD experimental concentration in the emulsions (mg/g)	mean value	SD	percentage variation compared to the mean value	percentage variation compared to the expected value
T0 LOTTO A		0,94			1,25	1,77		1,14			-0,79	3,89
		0,86			-7,32	-6,85		1,17			2,12	6,94
		0,87			-6,38	-5,90		1,18			2,89	7,74
T0 LOTTO B		0,94			0,90	1,42		1,20			4,62	9,55
		1,01	0,93	0,051	8,54	9,09		1,14	1,15	0,036	-0,99	3,68
		0,90			-2,86	-2,36		1,12			-2,81	1,77
T0 LOTTO C		1,00			7,01	7,56		1,16			0,76	5,51
		0,90			-3,45	-2,95		1,09			-5,66	-1,21
	0,93	0,95			2,31	2,83	1,10	1,15			-0,15	4,56
T28 LOTTO A		0,85			-7,53	-7,67		1,21			4,59	9,95
		0,90			-2,21	-2,36		1,17			0,99	6,17
		0,91			-1,50	-1,65		1,10			-4,6	0,3
T28 LOTTO B		0,96			4,18	4,01		1,18			2,31	7,56
		0,95	0,92	0,058	2,29	2,13		1,18	1,15	0,043	2,5	7,76
		1,01			8,91	8,74		1,15			-0,15	4,98
T28 LOTTO C		0,86			-6,46	-6,61		1,11			-3,65	1,29
		0,87			-6,11	-6,26		1,20			3,54	8,86
		1,00			8,44	8,26		1,09			-5,54	-0,7
